# Safety and Feasibility of Percutaneous Gastrostomy Placement in Patients on Antiplatelet Therapy

**DOI:** 10.31486/toj.20.0048

**Published:** 2021

**Authors:** Richard Tramel, Tyler Sandow, Daniel April, Vijay Ramalingam

**Affiliations:** ^1^ Department of Radiology, Ochsner Clinic Foundation, New Orleans, LA; ^2^ The University of Queensland Faculty of Medicine, Ochsner Clinical School, New Orleans, LA

**Keywords:** *Aspirin*, *clopidogrel*, *gastrostomy*, *hemorrhage*, *platelet aggregation inhibitors*

## Abstract

**Background:** Guidelines recommend the discontinuation of clopidogrel prior to gastrostomy tube placement. The aim of this study was to examine the safety and feasibility of performing radiologically inserted gastrostomy (RIG) tube placement in patients taking clopidogrel and/or aspirin.

**Methods:** We performed an institutional review board–approved retrospective analysis of the medical records for 237 consecutive patients following RIG tube placement secondary to dysphagia from August 2017 to January 2019. Antiplatelet medications and RIG type placement techniques (push vs pull) were compared with bleeding complications. Complications were categorized based on the Society of Interventional Radiology clinical practice guidelines. Of the 237 patients with RIG tubes placed, 77 patients were on antiplatelet therapy: 55 on single antiplatelet therapy and 22 on dual antiplatelet therapy. Of the 55 patients on single antiplatelet therapy, 26 were taking clopidogrel and 29 were taking aspirin.

**Results:** A total of 9 bleeding complications were observed. The most common complication was minimal bleeding or hematoma around the incision site (n=7). No statistically significant increase was seen in bleeding rates when comparing patients on any antiplatelet therapy regimen vs none (*P*=0.15), single antiplatelet therapy vs none (*P*=0.13), clopidogrel vs none (*P*=0.71), or dual antiplatelet therapy vs none (*P*=0.61). No significant increase in the bleeding complication rate was noted when comparing the aspirin-only regimen vs clopidogrel alone (*P*=0.34).

**Conclusion:** These findings suggest that the risk of bleeding complications is not increased in patients taking clopidogrel and/or aspirin prior to RIG tube placement.

## INTRODUCTION

Percutaneous gastrostomy tubes are often placed for dysphagia and risk of aspiration secondary to stroke.^1^ A 2017 update from the American Heart Association (AHA) stated that approximately 795,000 strokes occur annually in the United States,^2^ and 8% to 20% of stroke patients require either short- or long-term enteral tube feeding.^3^ Evidence shows that early tube feeding following stroke has a survival benefit.^4^ As a result, a large population of patients require gastrostomy tube placement.

Current (2014) AHA and American Stroke Association (ASA) recommendations for acute management of transient ischemic attack (TIA) and ischemic stroke include the use of single antiplatelet therapy (aspirin or clopidogrel) or dual antiplatelet therapy (aspirin and clopidogrel).^5^ Dual antiplatelet therapy with aspirin and clopidogrel has been shown to reduce the risk of recurrent stroke following acute TIA and minor ischemic stroke in the first 90 days.^6^ Patients who have had strokes are routinely placed on an antiplatelet therapy regimen because of these findings.

Endoscopic- and image-guided methods have been proven to be safe and effective for placement of percutaneous gastrostomy tubes.^7^ The current (2011) standard of practice for gastrostomy tube placement is based on multidisciplinary practical guidelines from the Society of Interventional Radiology (SIR) and the American Gastroenterological Association Institute.^8^ These guidelines include recommendations from both the American Society for Gastrointestinal Endoscopy (ASGE) and the SIR on radiologically inserted gastrostomy (RIG) tube placement for patients prescribed antiplatelet therapy. The ASGE considers RIG tube placement a high-risk procedure and recommends withholding clopidogrel for 7 days and replacing it with aspirin. The SIR considers RIG tube placement a moderate risk for hemorrhage and recommends withholding clopidogrel for 5 days but does not recommend withholding aspirin.

While studies have evaluated the safety of performing endoscopic gastrostomy tube placement in patients taking clopidogrel or dual antiplatelet therapy, as of 2020, little had been published in the radiologic literature evaluating the safety and feasibility of gastrostomy tube placement in the angiography suite in patients taking single or dual antiplatelet therapy with clopidogrel.

The purpose of this study was to evaluate the safety and feasibility of placing RIG tubes in patients taking single or dual antiplatelet therapy of aspirin and/or clopidogrel.

## METHODS

An institutional review board (IRB)–approved, Health Insurance Portability and Accountability Act–compliant retrospective review was conducted for all patients who had a RIG tube placed between August 1, 2017 and January 18, 2019 at a single institution. Informed consent was waived by the IRB. The Epic electronic medical record (Epic Systems Inc) was queried during the study dates for all patients who presented to interventional radiology for RIG tube placement. Inpatient and outpatient populations were included. Antiplatelet therapy regimen and RIG tube type (push vs pull) were collected.

Patients who received either aspirin within 24 hours or clopidogrel within 5 days of the procedure were categorized as having received antiplatelet therapy. Aspirin dosage was recorded. Patients were further categorized as either having received single antiplatelet therapy or dual antiplatelet therapy. The single antiplatelet therapy cohort included patients who were on aspirin or clopidogrel and had received the medications in the time frames listed previously. Patients on aspirin or clopidogrel alone were analyzed independently. The dual antiplatelet therapy cohort included patients who were on aspirin and clopidogrel.

Patients’ electronic medical records were reviewed for bleeding complications that occurred within 14 days following the procedure, and the bleeding events were categorized based on the SIR clinical practice guidelines.^9^ A bleed that required no intervention or nominal therapy, such as a bandage change, was categorized as a minor complication (type A or B). A bleed resulting in administration of blood products, procedural intervention, or increased length of hospital stay was categorized as a major complication (type C, D, E, or F). Mortality data were collected.

Statistical analysis was performed using JMP statistical software, version 15 (SAS Institute Inc). Chi-square and Fisher exact tests were used to assess for bias in the distribution of categorical variables. A *P* value <0.05 was deemed to be significant.

All percutaneous gastrostomy tube placements were performed by 1 of 7 fellowship-trained interventional radiologists, with experience ranging from 1 year to 27 years. Pull-type gastrostomies were 20 Fr EndoVive Standard Percutaneous Endoscopic Gastrostomy Kits (Boston Scientific Co). Push-type gastrostomies were Entuit Start Initial Placement Gastrostomy Sets (Cook Medical), ranging in size from 16 to 20 Fr. T-tacks sutures were used during the placement of push-type gastrostomies. Dilators were used for both push- and pull-type gastrostomies.

Prior to the procedure, all patients were placed on nothing by mouth status beginning at midnight before the procedure, and other anticoagulants were held. Heparin was held for 6 hours prior to the procedure, and enoxaparin sodium was held for 1 dose prior. Procedures were preferably performed with patients’ international normalized ratio (INR) ≤1.5 and platelet count >50,000 PLTs/mL; however, 1 patient had the procedure performed with an INR >1.5. No patients had platelet counts <50,000 PLTs/mL.

## RESULTS

We identified 237 patients who had gastrostomy tubes placed for dysphagia during the study interval. Sex distribution was equal, with 119 males and 118 females. Patient ages ranged from 23 to 99 years old, with a mean age of 67.9 years. One hundred forty-five patients (61.2%) received push-type gastrostomies, and 92 patients (38.8%) received pull-type gastrostomies (Table 1). All procedures were technically successful (100%), and no deaths occurred secondary to RIG tube placement.

**Table 1.** Patient Demographics and Clinical Information (n=237)

Variable	Value
Mean age, years (range)	67.9 (23-99)
Sex	
Male	119 (50.2)
Female	118 (49.8)
Radiologically inserted gastrostomy tube type	
Push	145 (61.2)
Pull	92 (38.8)
Any antiplatelet therapy	77 (32.5)
Single antiplatelet therapy	55 (23.2)
Aspirin alone	29 (12.2)
Clopidogrel alone	26 (11.0)
	
Dual antiplatelet therapy	22 (9.3)
Aspirin regimen	
81 mg	34 (14.3)
325 mg	17 (7.2)

Note: Data are presented as n (%) unless otherwise indicated.

A total of 77 patients (32.5%) were on antiplatelet therapy: 55 (23.2%) on single antiplatelet therapy and 22 (9.3%) on dual antiplatelet therapy. Of the 55 patients on single antiplatelet therapy, 29 patients were on aspirin alone and 26 were on clopidogrel alone.

Table 2 presents the counts and classifications of bleeding complications associated with RIG tube placement in the cohort. A total of 9 (3.8%) bleeding complications were observed, 7 of which were minor complications. Minor complications included minimal incision site hemorrhage (n=5, type B) or subcutaneous hematoma (n=2, type A), requiring no or nominal treatment. Five of the 7 patients with minor bleeding complications were on an antiplatelet therapy regimen. Two major bleeding complications (type D) occurred that required interventions and prolonged hospital stays. These bleeds resulted in a blood transfusion in one case and an esophagogastroduodenoscopy in the second. Both major complications occurred in patients who were not on antiplatelet therapy.

**Table 2.** Bleeding Complications Following Radiologically Inserted Gastrostomy Tube Placement (n=237)

Classification^a^	n (%)
Minor	7 (3.0)
A	2 (0.8)
B	5 (2.1)
Major	2 (0.8)
D	2 (0.8)
Overall	9 (3.8)

^a^Complications are categorized based on the Society of Interventional Radiology clinical practice guidelines.^9^ Minor complications (type A or B) are those that require no intervention or nominal therapy. Major complications (type C, D, E, or F) are those that result in administration of blood products, procedural intervention, or increased length of hospital stay.

Bleeding complications occurred in patients aged 51, 57, 69, 74, 79, 79, 81, 90, and 98 years. No specific age had a statistically significant increased bleeding risk (age range, 23 to 99 years, *P*=0.99). No significant increase in the bleeding complication rate was observed between the sexes (*P*=0.72). One minor bleeding complication occurred in the 1 patient with an INR >1.5. The patient's INR was 1.9.

As shown in Table 3, the comparison between the 5 patients on any antiplatelet therapy who had a bleeding complication vs the 4 patients with bleeding complications who were not on an antiplatelet therapy regimen was not statistically significant (6.5% vs 2.5%, *P*=0.15).

**Table 3.** Association of Bleeding Complications (n=9) With Demographic and Clinical Variables

	Number (Percentage)	
Variable/Comparison	With Bleeding Complications	*P* Value
Sex	4 (3.4) vs 5 (4.2)	0.72
Male (n=119) vs female (n=118)		
Radiologically inserted gastrostomy tube type		
Push (n=145) vs pull (n=92)	3 (2.1) vs 6 (6.5)	0.85
Antiplatelet therapy (n=77) vs no antiplatelet therapy (n=160)	5 (6.5) vs 4 (2.5)	0.15
Single antiplatelet therapy (n=55) vs dual antiplatelet therapy (n=22)	4 (7.3) vs 1 (4.5)	0.65
Aspirin (n=29) vs clopidogrel (n=26)	3 (10.3) vs 1 (3.8)	0.34
Aspirin (n=29) vs dual antiplatelet therapy (n=22)	3 (10.3) vs 1 (4.5)	0.43
Aspirin regimen		
81 mg (n=34) vs 325 mg (n=17)	2 (5.9) vs 2 (11.8)	0.47

No statistically significant increase in bleeding complications was observed when comparing patients on single antiplatelet therapy to patients who were not on any antiplatelet therapy regimen (7.3% vs 2.5%, *P*=0.13) or when comparing the dual antiplatelet therapy cohort to patients not on any antiplatelet therapy (4.5% vs 2.5%, *P*=0.61). Likewise, when comparing the dual antiplatelet therapy patients to the single antiplatelet therapy patients, no significant increase was observed (4.5% vs 7.3%, *P*=0.65).

**Figure.**
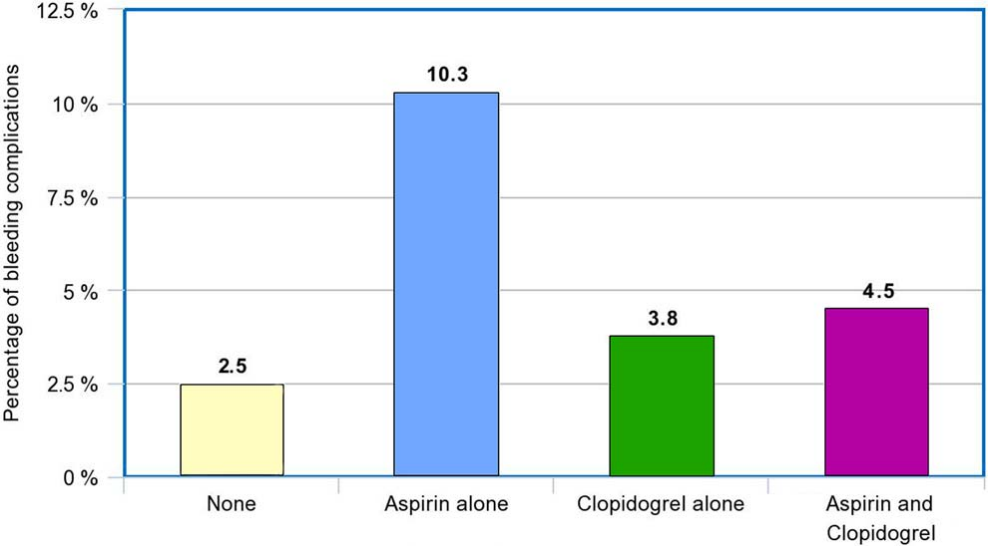
Bleeding complication percentages following gastrostomy tube placement. Patients who were not taking any antiplatelet therapy are labeled as none. Patients on dual antiplatelet therapy are labeled as aspirin and clopidogrel.

The Figure shows bleeding complication percentages by group. Minor bleeding complications were observed in 3 of 29 patients taking aspirin alone. No significant increase was observed when comparing patients on aspirin alone to those not on antiplatelet therapy (10.3% vs 2.5%, *P*=0.07) or when the aspirin-alone group was compared to the dual antiplatelet therapy group (10.3% vs 4.5%, *P*=0.43).

A total of 34 patients received an aspirin dose of 81 mg prior to RIG tube placement, and 17 received 325 mg. Two patients in each group had bleeding complications, but no significant increase in bleeding complication rates was observed in the comparison of aspirin doses (5.9% vs 11.8%, *P*=0.47).

One of the 26 patients on clopidogrel alone had a minor bleeding complication, but this finding was not statistically significant when compared to the bleeding complication rate of patients not on antiplatelet therapy (3.8% vs 2.5%, *P*=0.71). Further, no significant increases in bleeding rate were found when comparing the clopidogrel-alone cohort to the aspirin-alone cohort (3.8% vs 10.3%, *P*=0.34) or when comparing the clopidogrel-alone cohort to the dual antiplatelet therapy cohort (3.8% vs 4.5%, *P*=0.45).

Bleeding complications were encountered in 6 of 92 pull-type RIG tube placements vs 3 of 145 push-type RIG tube placements (6.5% vs 2.1%, *P*=0.85). Despite more incidences of bleeding complications with pull-type RIG tube placements, both of the major complications occurred in patients who received push-type RIG tube placements.

## DISCUSSION

Many people are on long-term aspirin therapy for prevention of cardiovascular disease.^10^ In this population, the use of clopidogrel and dual antiplatelet therapy is likely to increase given recommendations by the American College of Cardiology/AHA for patients with coronary artery disease,^11^ as well as recommendations by the AHA and ASA for the prevention of stroke in patients with prior stroke and TIA.^5^ Benefits of dual antiplatelet use have also been shown in patients with symptomatic peripheral arterial disease.^12^

Ischemic stroke patients can be a particularly difficult patient population to treat, given their antiplatelet therapy requirements and their need for long-term enteral access. While no increase in bleeding complications has been shown in patients who undergo endoscopically guided gastrostomy tube placement while on antiplatelet therapy,^13,14^ to our knowledge, this study is the first to evaluate the initial safety profile of RIG tube placement in the interventional radiology suite while on clopidogrel alone or dual antiplatelet therapy (clopidogrel and aspirin).

In this retrospective study, we found no difference in bleeding complication rates when comparing patients who were prescribed aspirin and/or clopidogrel vs those who did not use either medication prior to RIG tube placement for dysphagia. Similarly, we found no difference in bleeding complication rates between patients who were prescribed a single antiplatelet regimen (with aspirin or clopidogrel) vs patients who were prescribed a dual antiplatelet regimen (with aspirin and clopidogrel) prior to RIG tube placement for dysphagia. No difference in bleeding complication rates was observed when comparing patients taking clopidogrel vs aspirin prior to RIG tube placement. Also, while not statistically significant, the percentage of patients with a bleeding complication was lower in the groups of patients who were taking clopidogrel alone or dual antiplatelet therapy compared to patients taking aspirin alone.

The overall bleeding complication rate of 3.8% (9 of 237 patients) is higher than the expected rate of 0% to 2.5% noted in the SIR clinical practice guidelines.^8^ This higher-than-expected bleeding rate is believed to be secondary to our decision to use a broad definition of postprocedural bleeding, as the majority of our reported bleeding complications would likely be considered routine postprocedural changes. The point of using a broad definition was to ensure that any potential complications were reported in the final tabulation of the data. To further this point, all bleeding complications that occurred in patients in the antiplatelet therapy cohort were minor complications, per the SIR clinical practice guidelines, and required no intervention or nominal therapy, such as a bandage change. Practitioners should consider whether minor bleeding complications should have a major influence on clinical practice, as the benefits of continuing dual antiplatelet therapy or clopidogrel may outweigh the risks of minor bleeding complications.

This study has several limitations. The small sample size and retrospective nature of the study make broad applicability difficult; a robust patient cohort and prospective study are required to validate the findings. This study was performed at 1 institution, with 7 different physicians performing the procedure. Varying levels of skill among the physicians may have affected data outcomes. Further, clopidogrel response assays and P2Y12 tests were not performed prior to procedures.

## CONCLUSION

The results of this study suggest no differences in bleeding complication rates with RIG tube placement for dysphagia between patients receiving clopidogrel and/or aspirin and patients who are not taking antiplatelet therapy.

## Notes

*This article meets the Accreditation Council for Graduate Medical Education and the American Board of Medical Specialties Maintenance of Certification competencies for Patient Care and Medical Knowledge.*
